# Efficacy and safety of Tanreqing oral liquid in treatment of acute bronchitis: study protocol for a randomized controlled trial

**DOI:** 10.1186/s13063-022-06318-5

**Published:** 2022-05-07

**Authors:** Guilan Cheng, Bin She, Bing Mao, Hongli Jiang

**Affiliations:** 1 Department of Integrated Traditional Chinese and Western Medicine, West China Hospital, Sichuan University/West China School of Nursing, Sichuan University, 37 Guoxue Lane, Chengdu, 610041 Sichuan Province People’s Republic of China; 2 Department of Integrated Traditional Chinese and Western Medicine, Division of Respiratory Medicine, West China Hospital, Sichuan University, 37 Guoxue Lane, Chengdu, 610041 Sichuan Province People’s Republic of China

**Keywords:** Acute bronchitis, Tanreqing oral liquid, Traditional Chinese medicine, Randomized controlled trial, Study protocol

## Abstract

**Background:**

Approximately 5% of adults have an episode of acute bronchitis each year, accounting for more than 10 million medical visits yearly. The primary goal of treatment is reduction of symptoms. Currently, available medications are questionable in effectiveness and safety and are not recommended for routine use in clinical practice. Although Chinese herbal medicine has been widely used in the management of acute bronchitis in China, evidence-based data is lacking. This trial aims to evaluate the efficacy and safety of Tanreqing oral liquid in the treatment of acute bronchitis with phlegm-heat obstructing lungs syndrome.

**Methods/design:**

This study is a prospective, multi-center, randomized, double-blinded, parallel-group, placebo-controlled trial. A total of 270 acute bronchitis adult patients with phlegm-heat obstructing lungs syndrome will be enrolled from outpatients and emergency departments at nine study centers across China. All included patients will be randomly allocated to receive Tanreqing oral liquid or placebo oral liquid, 20 mL three times daily for seven consecutive days. The primary outcome will be cough resolution rate. Secondary outcomes will include change of bronchitis symptoms scores from baseline to post-treatment, cough relief rate, time to cough resolution, time to cough relief, resolution rate of a single symptom, combination medicine use, change of traditional Chinese medicine syndrome score from baseline to post-treatment, and adverse events.

**Discussion:**

This trial may provide an alternative treatment option for acute bronchitis patients, especially those in outpatients and emergency departments. It may also add evidence to Chinese herbal medicine for treating acute bronchitis.

**Trial registration:**

Chinese Clinical Trial Registry ChiCTR2000040264. Registered on 26 November 2020

**Supplementary Information:**

The online version contains supplementary material available at 10.1186/s13063-022-06318-5.

## Background

Acute bronchitis (AB) is a common clinical condition characterized by acute inflammation of the trachea and lower airways. About 95% of uncomplicated AB patients are caused by viral infections, and bacterial infections are not common [1, 2]. Approximately 5% of adults have an episode of AB per year, accounting for more than 10 million medical visits each year [3]. Patients predominantly present with cough with or without sputum production without evidence of pneumonia. Cough usually lasts no more than 3 weeks and is the major reason for both outpatients consultations and emergency departments attendances.

Although AB is considered to be self-limiting in most cases, symptomatic treatment is primarily required, particularly improving cough. Antibiotic agents have been widely but unnecessarily used worldwide. However, antibiotics use can only provide a modest reduction of cough duration by 0.46 days, and unnecessary antibiotics use may be associated with high antibiotic cost, increased antibiotic resistance, and potential side effects [4]. Therefore, antibiotics are not recommended for routine use in uncomplicated AB patients [5, 6]. Similarly, antiviral therapy, inhaled corticosteroids, inhaled beta agonists, inhaled anticholinergics, oral corticosteroids, antitussives, oral non-steroidal anti-inflammatory drugs, or alternative therapies are also not recommended for routine use in clinical practice for effectiveness and safety concerns [3, 7].

Chinese herbal medicine, an important part of complementary and alternative medicine, has been widely used in the management of AB [8]. However, evidence-based data is still lacking. Tanreqing oral liquid is a new sixth category oral drug of traditional Chinese herbal medicine jointly developed by Henan Xinyi Pharmaceutical Co., Ltd., and Beijing Jifatang Institute of Chinese Medicine. It is primarily composed of Huangqin (Radix Scutellariae Baicalensis), Xiongdanfen (Fel Selenarcti), Shanyangjiao (Cornu Naemorhedi), Jinyinhua (Flos Lonicerae), and Lianqiao (Forsythiae Fructus) and has the effects of clearing heat, detoxifying, resolving phlegm, and suppressing cough and has anti-infection and anti-inflammatory potential [9, 10]. Tanreqing oral liquid is an extension product of Tanreqing injection, which has been widely used in inpatients and emergency patients with bronchitis or pulmonary infections in China [11, 12].

This prospective, multi-center, randomized, double-blinded, parallel-group, placebo-controlled, superiority trial aims to evaluate the efficacy and safety of Tanreqing oral liquid versus placebo oral liquid in the treatment of AB with phlegm-heat obstructing lungs syndrome.

## Methods/design

This study has been authorized by China State Food and Drug Administration (Approval No. 2005L02403) and was registered with the Chinese Clinical Trial Registry (ChiCTR2000040264). The current study protocol (version 3.0, date 30 August 2019) has been reviewed and approved by the Biomedical Ethics Committee of West China Hospital, Sichuan University (Chengdu, China) on 13 April 2020. This study is sponsored by Shanghai Kaibao Pharmaceutical Co., Ltd. (Shanghai, China); however, the sponsor is not involved in the study design, data collection, data management, future data analysis, or data interpretation.

### Patient population and setting

**Fig. 1:**
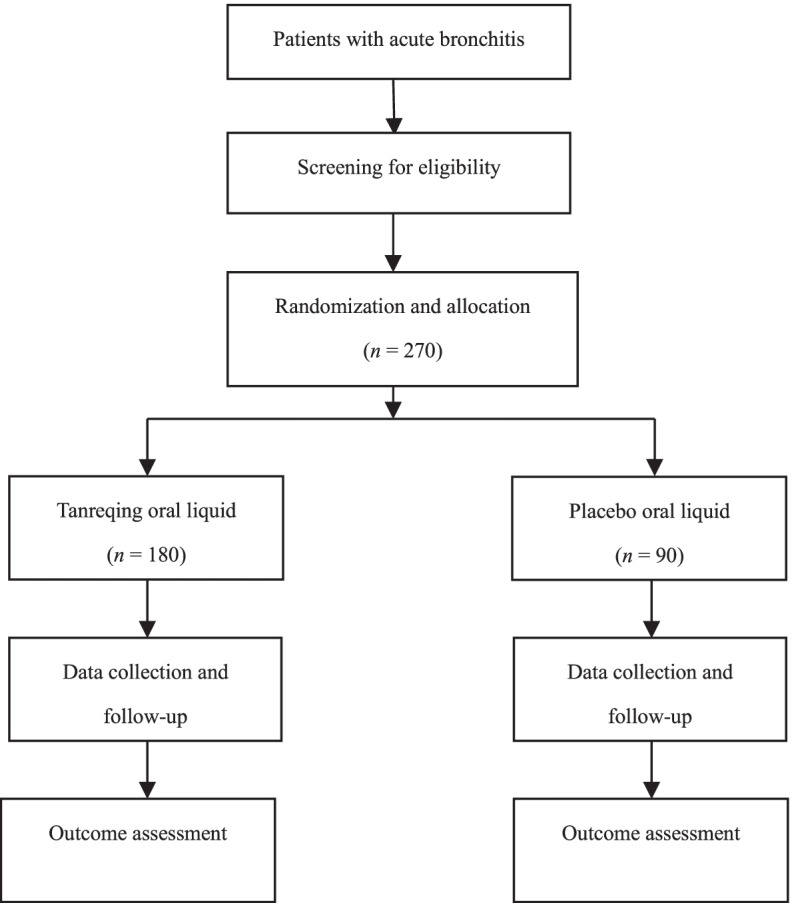
Study flow chart

In this trial, AB will be clinically diagnosed based on the predominant cough with or without sputum production, lasting no more than 3 weeks, but with no evidence of medical history, lung exam, or imaging findings suggesting common cold, pneumonia, acute exacerbation of asthma, bronchiectasis, or chronic obstructive pulmonary disease [13]. The traditional Chinese medicine (TCM) diagnosis of phlegm-heat obstructing lungs syndrome will be made according to the Guidelines for Clinical Research of New Chinese Medicine and TCM guideline for AB [14, 15]. The detailed criteria are listed in Table 1. A total of 270 patients will be enrolled from nine study centers across China: (1) West China Hospital of Sichuan University (2) the First Affiliated Hospital of Guizhou University of TCM (3); Ruikang Hospital Affiliated to Guangxi University of Chinese medicine (4); Chinese Medicine Hospital of Shanghai, (5) the First Affiliated Hospital of Hunan University of Chinese medicine (6); Central Hospital of Minhang District, Shanghai (7); Yueyang Hospital of Integrated Traditional Chinese and Western Medicine, Shanghai University of TCM (8); Guangdong Provincial Hospital of Chinese medicine; and (9) Second Affiliated Hospital of Tianjin University of TCM. Eight of them are academic/teaching hospitals. A research assistant in each study center will recruit 30 patients from the outpatients and emergency departments. Patient recruitment is scheduled to begin from November 2020 to December 2021. Figure 1 shows the flow chart of the trial.

**Table 1:** Diagnostic criteria for phlegm-heat obstructing lungs syndrome

Category	Symptoms or signs
Main symptoms	Cough, expectoration
Minor symptoms	Fever; sore throat; chest tightness; thirst; yellow urine; dry stool
Tongue	Red tongue with a yellow or yellow greasy coating
Pulse condition	Slippery and rapid pulse

Diagnostic criteria: both main symptoms + at least one of the minor symptoms + appropriate tongue and pulse condition

AB patients aged 18 to 65 years old, with disease course within 72 h, scoring ≥ 6 points in bronchitis severity score, scoring ≥ 4 points in total cough symptom score, with expectoration in TCM symptom score of 6 points or more, will be included in this trial. Patients who have respiratory diseases such as chronic obstructive pulmonary disease, bronchiectasis, asthma, lung cancer, tuberculosis, pneumonia, and lung abscess; who have severe underlying cardiac, cerebral, hematological, hepatic, or renal disorders or other diseases significantly affecting the survival and prognosis, such as cancer; who have used other medicines after disease onset including antibiotics, expectorants, antitussives, systemic or inhaled corticosteroids, inhaled bronchodilators, and similar Chinese medicines; who use an angiotensin-converting enzyme inhibitor in the last 1 month; who is current smokers or recent ex-smokers quitting smoking less than 1 month ago; whose body temperature is 38.5 °C or higher; or whose ALT or AST > 1.5 times of normal upper limit, urine protein >+, serum creatine abnormality, white blood cell count < 3 × 10^9^/L or > 10 × 10^9^/L, and/or neutrophil granulocyte > 80% will be excluded from the trial. The detailed inclusion and exclusion criteria are listed in Table 2.

**Table 2:** Patients’ inclusion and exclusion criteria

**Inclusion criteria**	
1. Diagnosis of acute bronchitis	
2. Phlegm-heat obstructing lungs syndrome in traditional Chinese medicine Zheng	
3. Total cough symptom score ≥ 4 points	
4. Expectoration in traditional Chinese medicine symptom score ≥ 6 points	
5. Bronchitis severity score ≥ 6 points	
6. Disease course within 72 h	
7. Aged 18 to 65 years old	
8. Voluntarily provide written and informed consent	
**Exclusion criteria**	
1. Patients with respiratory diseases such as chronic obstructive pulmonary disease, bronchiectasis, asthma, lung cancer, tuberculosis, pneumonia, lung abscess, and chest X-ray showing lung inflammation lesions	
2. Patients with severe underlying cardiac, cerebral, hematological, hepatic, or renal disorders or other diseases significantly affecting the survival and prognosis, such as cancer	
3. Current smokers or recent ex-smokers quitting smoking less than 1 month ago	
4. Use of an angiotensin-converting enzyme inhibitor in the last 1 month	
5. Patients with body temperature ≥ 38.5 °C	
6. ALT or AST > 1.5 times of normal upper limit, urine protein >+, serum creatine abnormality, white blood cell count < 3 × 10^9^/L or > 10 × 10^9^/L, and/or neutrophil granulocyte > 80%, or those who need antibiotic therapy	
7. Those who have used other medicines after disease onset including antibiotics, expectorants, antitussives, systemic or inhaled corticosteroids, inhaled bronchodilators, Chinese medicines of relieving exterior syndrome with pungent-cool drugs, clearing away heat, and detoxification and other related Chinese medicines	
8. Pregnant or lactating women and those who have pregnancy plans in the last 3 months	
9. Allergic constitution or known to be allergic to any ingredients in tested drug	
10. Mental patients or legal disability	
11. Patients who have participated or are currently participating in another clinical trial in the last 1 month	
12. Patients who are inappropriate for participation judged by researchers	

### Withdrawal and termination criteria

Researchers can withdraw participants for the following reasons: (1) patients’ body temperature rises to 39 °C or more suggesting that other treatments may be needed (2); patients’ symptoms significantly worsen after three treatment days, in this case, trial drugs may need to be stopped and other treatments may need to be provided (3); patients develop serious adverse events (SAEs) or allergic reaction (4); patients accompany with severe comorbidities or complications (5); patients use forbidden medications; and (6) blindness is broken or need to be uncovered urgently for certain reasons. Participants themselves can voluntarily withdraw from the study at any time for any reason. In this case, researchers will make efforts to know and record their withdrawal reasons as much as possible, such as poor efficacy, intolerable to certain adverse reactions, economic factors, lost to follow-up without explanation, or other reasons for dropout.

National Pharmaceutical Supervisory and Administrative Department can terminate the whole trial for any reason. The primary investigators may terminate or suspend the study due to SAEs, poor efficacy, or significant deviation from the protocol during the study. The sponsors can also terminate the study because of SAEs or poor efficacy.

### Randomization and blinding

Central stratified block randomization method will be used in this trial. Two columns of 270 random arrangements of treatments (trial drugs and placebo drugs ratio of 2:1) will be generated by an independent statistician using the Statistical Analysis System (SAS 9.4). The treatment allocation table will be completed after blinding, corresponding to the sequential numbers 001 to 270. An appointed drug administrator will issue drugs according to the drug number. The blinding process should be recorded in writing and signed by all blinding personnel. Actual treatment allocation of trial drugs and placebo drugs will be sealed and stamped on the spot, in duplicate, and saved by the drug clinical trial institution and the sponsor. Trial participants, investigators, and research assistants together with statisticians will be all blinded to treatment allocation throughout the study. If there are serious complications or adverse events during the trial, which affect the trial conduct and the choice of treatment measures, the blind can be broken urgently with the consent of the primary investigator. The reason, time, place, and signature of uncovering the blind will be recorded in detail.

### Interventions

**Fig. 2:**
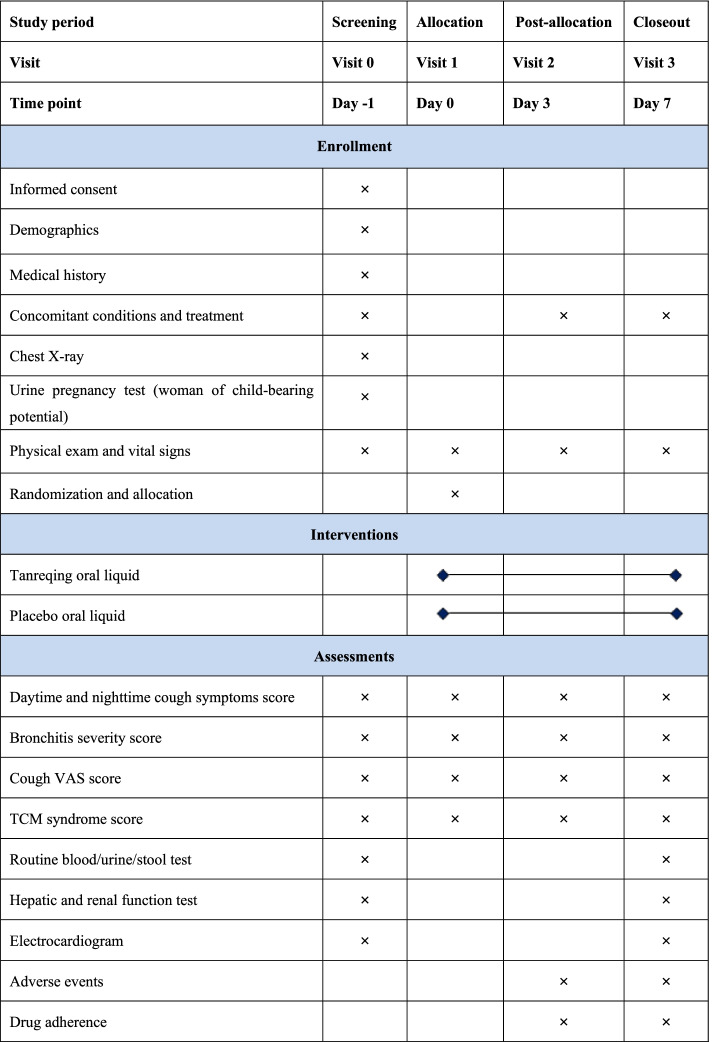
Schedule of study procedures. TCM, traditional Chinese medicine; VAS, visual analog scale

All patients will be randomly allocated to either the Tanreqing group or the placebo group. Patients in either group will receive Tanreqing oral liquid or placebo liquid, 20 mL three times daily for seven consecutive days. Figure 2 provides the details of the study procedures.

#### Concomitant treatments and forbidden medication

Medications used to control the underlying diseases such as hypertensive or diabetic mellitus, will be permitted, but newly use of angiotensin-converting enzyme inhibitors will not be allowed. Chinese medicine and other Western medicine that may relieve cough or sputum will not be permitted for potential impact on the efficacy and safety analysis. Antibiotics, expectorants, or antitussives can be taken in combination only when patients’ conditions significantly worsen. All used medications should be recorded in the original research medical records, including name, dose, mode of administration, and treatment duration.

#### Intervention adherence and compliance

Researchers will take the following measures to improve adherence to the intervention protocol: (1) sufficiently implementing informed consent, informing the potential benefits and risks, and instructing patients to fully understand the importance of taking medication according to the study protocol before participation (2); recording contact details of participants to keep in touch, including but not limited to mobile phone, QQ, WeChat, and email (3); highlighting the visit time in patient diary card; and (4) calling to remind the participants 1 day before a visit. At the final visit, patients will be requested to bring back the remaining drugs and packaging and be asked whether they take the drugs in the correct amount on time, whether they miss the medication, or whether they take fewer or more medications. All the information should be recorded in the original research medical records in a timely manner. Trial drug compliance will be calculated as the actual taken amount divided by the total scheduled amount.

### Outcome measures

#### Primary outcome

Cough resolution rate: proportion of patients whose cough is completely resolved after treatment.

The overall frequency, severity, and impact of cough will be evaluated using a patient self-rated cough symptom score (CSS) scale. CSS scale will be graded twice daily at two intervals: daytime (from 8:00 am to 8:00 pm) and nighttime (from 8:00 pm to 8:00 am), each scored from 0 to 3 points [16]. Cough resolution will be defined as both daytime and nighttime CSS scale reduced to 0 without relapse for two consecutive days.

#### Secondary outcomes

The following are the secondary outcomes:

1. Change of bronchitis severity scores from baseline to post-treatment: Bronchitis severity score (BSS) is an observer-rated tool for assessing the severity of five major symptoms of AB including cough, sputum production, rales/rhonchi (auscultation), chest pain during coughing, and dyspnea, each rating from 0 to 4 points. The BSS is the sum of the five ratings ranging from 0 to 20 points [17].
2. Cough relief rate: Cough relief will be defined as both daytime and nighttime CSS decreasing to 1 or less, or decreasing by 1 and lasting for two consecutive days. Cough relief rate will be the proportion of patients who achieve cough relief after treatment.
3. Time to cough resolution: length of time at baseline to cough resolution: For example, the daytime and nighttime CSS on the patient’s diary cards on the 5th and 6th days are all 0 points, then the cough resolution time is recorded as “5 days.”
4. Time to cough relief: Length of time at baseline to cough relief.
5. Resolution rate of single TCM symptom.
6. Combination medication use: Combination medication here will only include antibiotics, expectorants, and antitussives.
7. Change of TCM syndrome score from baseline to post-treatment: Each TCM symptom will be graded (see Additional file 1). TCM syndrome score will be the sum of the main symptom score and all minor symptoms scores.
8. Adverse events: Besides routine blood, urine, and stool tests and hepatic and renal functions, any unexpected symptom or sign during the treatment period will be recorded and analyzed.

### Coordination and conduct of the trial

A multicenter trial coordination committee will be established and responsible for trial conduct. Data monitoring committee will not be established because (1) this trial will recruit AB participants and perform follow-up over a short period of 7 days, (2) pilot clinical observation found Tanreqing oral liquid was well tolerated and safety data from Tanreqing injection did not show any toxic potential in real-world cohorts [18], and (3) the primary outcome measure will be patients self-reported resolution of symptoms. Similarly, interim safety and efficacy analysis will not be conducted. An independent monitoring committee will conduct onsite monitoring monthly in accordance with the sponsor’s requirements and relevant regulations. An independent qualified auditor will randomly audit the trial to evaluate trial conduct and compliance with the protocol, good clinical practice, standard operating procedures, and the applicable regulations.

### Data collection and management

Research medical records will be the original documents in this trial, which will be properly kept by each study center. Trained researchers will collect baseline demographic parameters, outcome data, and other trial data at each visit. All participants will be instructed to grade their cough and TCM symptoms and record medication use and any unexpected condition on the daily diary card.

Plans to promote participant retention will be as follows: (1) instructing participants to fully understand the importance of study involvement and acknowledging their participations (2); recording contact details of participants to keep them in touch, including mobile phone, QQ, WeChat, and email (3); calling to remind participants one day before a scheduled visit (4); trying to re-contact or re-schedule at their convenient time in case of a missed visit (5); trying to collect outcome data when a participant withdraws from the study; and (6) providing a compensation of ¥600 after completing follow-up.

In this study, an electronic case report form (ECRF) created by electronic data acquisition system (EDC) will be used to collect and manage data online. The specific procedures and processes of data management will be standardized in data management plan to ensure real-time, traceability, consistency, accuracy, and integrity of data. The data entry personnel designated by each center will log into EDC, timely input related data at each visit. Data verification methods will include (1) on-site source data verification: a designated clinical supervisor will check the consistency of ECRF data and source data (2); system automatic logic verification: EDC will conduct automatic logic verification at the same time of data input and send out system questions in real time; and (3) manual verification: an independent data manager will manually check the data and issue manual queries if there is a problem. Researchers or researchers’ designated personnel will answer queries online in real time, and data administrators will review the answers, confirm to close, or issue questions again until the data is “clean.” At the end of the trial, the documents generated in the data management process will be saved and filed according to good clinical practice, requirements. Only data managers and statisticians will have access to exporting the final data. The sponsor and each center will store the ECRF in PDF format in a durable storage medium that cannot be edited.

### Adverse event reporting

An adverse event (AE) will be any unexpected or undesirable experience or condition throughout the treatment period, including clinical symptoms or signs irrelevant to AB, and abnormal laboratory findings. All AEs will be recorded in detail including specific presentation, occurrence date, degree, duration, the process, treatment measures, clinical outcome and prognosis. The causal relationship between an AE and trial drug will be timely judged according to the general guidelines for clinical research of new drugs of traditional Chinese medicine (November 2015) issued by China State Food and Drug Administration. A SAE will be defined as any adverse medical event that occurred at any dose at any time throughout the whole trial. SAEs may (1) result in death, (2) be life-threatening, (3) result in persistent or significant disability or incapacity, (4) require inpatients hospitalization or extend the length of hospital stay, or (5) be other important medical events in which the abovementioned conditions may occur without treatment. Any SAE must be reported to the Biomedical Ethics Committee of West China Hospital of Sichuan University, the sponsor, and China’s State Food and Drug Administration within 24 h.

### Sample size calculation

The sample size was calculated based on the comparison of cough resolution rate between the Tanreqing oral liquid group and the placebo group. According to the pilot study and expert consultations, the cough resolution rate for the Tanreqing oral liquid group and the placebo group was estimated as 80% and 40%, respectively, with a difference of 20% or higher considering clinically significant. According to the formula of ${n}_c=\frac{{\left({z}_{1-\alpha }+{z}_{1-\beta}\right)}^2}{{\left({\pi}_T-{\pi}_C-\Delta \right)}^2}\left[\frac{\pi_T\left(1-{\pi}_T\right)}{K}+{\pi}_C\left(1-{\pi}_C\right)\right]$, where *π*_*T*_, *π*_*C*_ were the estimated cough resolution rate for the trial and placebo group of 0.8 and 0.4, respectively, Δ was the superior margin of 0.2, *α* of 0.025 was the one-sided 0.025 level of significance, *β* of 0.2 was the type II error, and the sample size of the placebo group was 63. According to the Tanreqing oral liquid group to placebo group ratio of 2:1, the sample size of the Tanreqing oral liquid group was 126.

According to regulatory requirements of a new 6th category oral drug of traditional Chinese medicine, the overall number of subjects in the Tanreqing oral liquid group should be no less than 300. Meanwhile, there are two independent clinical trials designed for different indications, i.e., AB and the common cold respectively to evaluate the efficacy and safety of Tanreqing oral liquid. Therefore, 300 subjects were divided into each indication equally to ensure that the overall subjects in Tanreqing oral liquid group would satisfy the regulatory requirement. Therefore, the total sample size was 225 (150/75 for the treatment and control group, respectively). Considering an expected post-randomization dropout rate of 20%, the sample size was set as 270, with 180 for Tanreqing oral liquid group and 90 for the placebo group.

### Statistical analysis

The full analysis set (FAS) population will include all randomized participants who receive at least 20 mL trial medication and collect outcome data after treatment. It will be used to describe the analysis set which is as complete as possible and as close as possible to the intention-to-treat ideal of including all randomized participants. Per-protocol (PP) population will include all randomized patients who strictly follow the protocol. The safety set (SS) population will include all randomized participants who receive at least 20 mL trial medication and have safety evaluation data. Missing efficacy data will be imputed using the last observation carried forward method. Efficacy and safety analysis will be performed based on the FAS and PP population and SS population.

Statistical analysis will be performed using SAS 9.4 according to the statistical analysis plan. The primary outcome of cough resolution rate will be analyzed using the chi-squared or Fisher’s exact tests. Furthermore, logistic regression will be conducted to provide a risk ratio. For secondary outcomes, *t* test and nonparametric tests will be used for quantitative data, and chi-squared or Fisher’s exact tests will be used for qualitative data. A one-sided *P*-value of 0.025 or less will be considered statistically significant.

### Ethical considerations and dissemination

Each study center will designate a research assistant to recruit patients from respiratory outpatients or emergency departments. After approval of the ethics committee, if there are significant changes to eligibility criteria, outcomes, and data analyses, the primary investigator will write and sign the “protocol modification specification,” and report to the Ethics Committee for approval again before implementation. Trained research investigators will explain detailed information regarding the study aim, potential benefits and risks, alternative treatment options, and patients’ rights and obligations to potential participants prior to participation. After obtaining voluntarily signed informed consent from participants, the research investigators will arrange some physical and laboratory examinations according to the study procedure to confirm whether participants really meet the inclusion and exclusion criteria. Then, research investigators will give a detailed introduction of the study protocol and study procedures to all eligible participants. Participants who might have difficulty in weighing the risks and benefits of the study will be excluded from this study. All participants will be free to refuse to take part in the trial and drop out of the trial during the process at any time. If patients who comply with the trial protocol suffer from any AE and need to be treated, the investigators will provide timely and effective therapy options and perform follow-ups until patients reach a complete resolution. Moreover, only researchers, monitors, drug regulatory departments, and ethics committee have access to participants’ personal medical records. Data processing will omit the information that can identify the individual identity. Each participant will be identified only with a unique subject identity never be disclosed to anyone in the result dissemination and publication.

The primary investigators and sponsor will communicate the trial results to participants and all the investigators involved. The trial results will be submitted to national or international conferences or peer-reviewed journals with the sponsor’s permission.

## Discussion

A systematic review [8] of 49 randomized controlled trials suggested that Tanreqing injection may improve effective rates, shorten the time to resolution of fever and cough, and was safe in the treatment of AB. However, none of the included trials was placebo-controlled, and the methodological quality of most studies was poor. Moreover, the majority population included were hospitalized patients. Tanreqing oral liquid is developed for oral administration and is an extension product of Tanreqing injection. This prospective, multi-center, randomized, double-blinded, parallel-group, placebo-controlled trial aims to investigate the efficacy and safety Tanreqing oral liquid in the treatment of AB (phlegm-heat obstructing lungs syndrome) in outpatients or emergency settings. It may provide an alternative option to AB patients in outpatients and emergency departments settings, and may add evidence to Chinese herbal medicine for treating AB.

There are some limitations to this protocol. In this trial, the diagnosis of AB will be established based on clinical symptoms and signs since there is no gold standard test available. However, X-ray will be performed to rule out pneumonia. Patients with chronic respiratory diseases including chronic obstructive pulmonary disease, bronchiectasis, asthma, lung cancer, and tuberculosis will be excluded. In addition, the microbiological test will not be carried out because nearly 95% of AB cases are caused by a viral infection, and antibiotics are not suggested by several international guidelines [5, 6, 19]. Furthermore, the microbiological test is not routinely performed in clinical practice in the outpatient settings.

### Trial status

The study protocol is version 3.0 on 30 August 2019. Patient recruitment began on 30 November 2020. Patient recruitment is scheduled to begin from 30 November 2020 to 31 December 2021.

## Supplementary Information

**Additional file 1.** Traditional Chinese medicine symptoms grading criteria.

## Data Availability

Not applicable.

## Abbreviations

AE: Adverse event
AB: Acute bronchitis
BSS: Bronchitis severity score
CSS: Cough symptom score
ECRF: Electronic case report form
EDC: Electronic data acquisition system
FAS: Full analysis set
PP: Per-protocol
SAE: Serious adverse event
SS: Safety analysis
TCM: Traditional Chinese medicine
