# Supplementary material for: Efficacy and safety of Tanreqing oral liquid in treatment of acute bronchitis: study protocol for a randomized controlled trial
Source: Trials. 2022 May 7;23:373. doi: 10.1186/s13063-022-06318-5 (PMC9077961; doi:10.1186/s13063-022-06318-5)
Supplement: Supplementary file 1 — Additional file 1. Traditional Chinese medicine symptoms grading criteria. [file 13063_2022_6318_MOESM1_ESM.pdf]

**Main symptoms**	**Score grading**			
	**0**	**3**	**6**	**9**
Cough	Not at all	Intermittent cough during the day	Coughing in the day or occasionally coughing at night, able to adhere to work	Frequent cough in the day and night, disturbing work and sleep
Expectoration	Not at all	A small amount of yellow phlegm	Moderate amount of yellow phlegm	Large amount of yellow phlegm
**Minor symptoms**	**Score grading**			
	**0**	**1**	**2**	**3**
Fever	Not at all	Conscious fever, ＜37.2℃	37.3-37.9℃	≥ 38.0℃
Sore throat	Not at all	Mild	Moderate	Severe
Chest tightness	Not at all	Mild	Moderate, tolerable	Significant, disturbing sleep or activity
Thirst	Not at all	Mild	Moderate	Severe; drinking more water
Dry stool	Not at all	Mild dry stool	Moderate dry stool	Constipation and abdominal Distension, need cathartics [illegible]
Deep-colored urine	Not at all	Yellow urine	Yellow and red urine, few amount	Brown urine, burning sensati[illegible] during urination
